# Latitudinal variation in seasonal migration of Eurasian curlews *Numenius a. arquata* breeding in Great Britain: breeding failure advances departure timing in southern populations

**DOI:** 10.1186/s40462-026-00683-5

**Published:** 2026-07-23

**Authors:** Aisha C. Bründl, Christopher J. Heward, Samantha E. Franks, Ryan A. Burrell, Harry Ewing, Eleanor M. Rivers, David Scott, Peter M. Potts, Katharine M. Bowgen, Andrew N. Hoodless

**Affiliations:** 1 Game & Wildlife Conservation Trust, Burgate Manor, Fordingbridge, Hampshire, SP6 1EF UK; 2 British Trust for Ornithology, The Nunnery, Thetford, Norfolk, IP24 2PU UK; 3 Devon & Cornwall Wader Ringing Group, Ilfracombe, Devon, UK; 4 Curlew Recovery Partnership, Burgate Manor, Fordingbridge, Hampshire, SP6 1EF UK; 5 School of Biological Sciences, University of East Anglia, Norwich Research Park, Norwich, Norfolk, NR4 7TJ UK; 6 Farlington Ringing Group, c/o Solent Court Cottage, Chilling Lane, Warsash, Southampton, Hampshire, SO31 9HF UK; 7 British Trust for Ornithology Cymru, Deiniol Road, Bangor, Gwynedd, LL57 2UW UK

**Keywords:** Conservation ecology, Eurasian curlew *Numenius arquata arquata*, GPS tracking, Movement, Reproductive success, Sex differences, Waders

## Abstract

**Background:**

Migration plays a crucial role in the annual cycle of migratory birds. The Eurasian curlew *Numenius arquata arquata*, a species of high conservation concern in Great Britain, exhibits a wide range of migratory behaviours across its western distribution, from resident and short-distance movements to long-distance migrations along the East Atlantic Flyway. However, migratory behaviours of British-breeding curlews remain poorly studied. In particular, the ecological and life-history factors underlying variation in migration, such as timing and duration, remain unclear in this declining species.

**Methods:**

We used GPS-tracking data from 80 Eurasian curlews breeding across a 703-km latitudinal gradient in Great Britain to examine how migration behaviours are influenced by sex, breeding-site latitude, local weather conditions, individual repeatability and reproductive success. Statistical analyses assessed relationships between these variables and timing of migration events.

**Results:**

We found various sex-specific migration behaviours indicating contrasting selection pressures. Curlews breeding at higher latitudes (Scotland and northern England) left wintering sites earlier and departed breeding sites later than birds breeding at lower latitudes (southern England), although both groups arrived at breeding sites at similar times. Breeding-site latitude also affected migration distance, duration and speed in both pre- and post-breeding migration. Although migration timing was consistent within individuals across years, timing also varied with local weather conditions. Warmer temperatures were associated with earlier pre-breeding departures and arrivals, whereas post-breeding movements occurred later under warmer, wetter and calmer conditions. Timing of post-breeding migration was related to reproductive outcomes: successful breeders departed later, likely reflecting parental care of broods. This effect was driven by southern birds, which showed overall lower breeding success and departed earlier than northern birds.

**Conclusions:**

Our main results reveal clear individual and latitudinal variation in curlew migration behaviour, with reproductive success influencing migration timing. These findings underscore the need to integrate life-history context into migration research, as spatial variation in breeding success, influenced by land-use and management, and climate change, can produce systematic differences in migratory timing. Such differences may influence subsequent habitat use across the annual cycle, with potential consequences for population dynamics and conservation of declining migratory wader species such as Eurasian curlews.

**Supplementary Information:**

The online version contains supplementary material available at https://doi.org/10.1186/s40462-026-00683-5.

## Background

Migration plays a fundamental role in the life histories of many bird species, affecting survival, reproductive success and population dynamics across the annual cycle [1, 2]. Because migration is energetically costly and exposes individuals to environmental risks, birds must balance the benefits of timely arrival with the energy expenditure and hazards associated with long-distance travel [3–5]. Optimal migration theory predicts that individuals minimise travel time and energy while maximising fitness by adjusting flight speed, timing and routes in response to environmental conditions such as wind, temperature and precipitation [6–9]. By fine-tuning these decisions, birds can improve migration efficiency, reduce energy costs and enhance survival and reproductive success.

The timing of arrival at breeding sites is a key component of migration decisions, as early arrival is often positively correlated with reproductive success [10]. Individuals arriving early can secure higher-quality territories and nesting sites, establish or re-establish pair bonds and increase opportunities for replacement clutches [2, 4, 11–14]. Early arrival may also reduce the risk of phenological mismatch between peak food availability and chick hatching, an issue of growing importance under climate change [15–17]. However, arriving too early can expose birds to adverse environmental conditions, highlighting a trade-off between the benefits of early arrival and the potential fitness costs associated with unpredictable weather [2, 18, 19]. These trade-offs can vary across environmental gradients, particularly latitude, where breeding seasons differ in duration and predictability. Birds breeding at higher latitudes often experience compressed reproductive windows and may therefore adopt faster or more direct migration behaviours, whereas populations at lower latitudes may have greater flexibility in migration timing. Such spatial structuring can produce distinct population-level migration behaviours, including leap-frog, telescopic and chain migration, reflecting the combined influence of environmental gradients, competition and population density on migratory behaviours [2, 20–25].

The Eurasian curlew *Numenius arquata* (hereafter curlew) exemplifies how environmental gradients, reproductive constraints and migratory behaviours interact across large spatial scales. Curlews breed across a broad latitudinal range spanning Europe and temperate Asia and migrate along several major flyways, including the East Atlantic Flyway. Previous studies using ringing recoveries and GPS tracking have shown that migration behaviours in curlews are spatially structured across this range [26–28]. In continental European populations, migration distance and duration increase with breeding latitude, with southern individuals generally arriving earlier and spending longer periods at their breeding sites [29].

This study focuses on *N. a. arquata* breeding in Great Britain. Great Britain supports an estimated breeding population of approximately 58,000 pairs [30], primarily in upland habitats such as rough pastures and moorlands in Scotland and northern England [31]. After breeding, many British curlews migrate to coastal habitats within Great Britain and Ireland for the winter, where they are joined by individuals from continental Europe, particularly Scandinavia, Finland and Russia, forming a wintering population of approximately 155,000 individuals [27, 30, 32, 33]. Despite evidence of spatial structuring in migration across continental populations, migration behaviours and their ecological drivers remain less well understood for curlews breeding in Great Britain, particularly regarding how breeding outcomes influence migration behaviour. This is especially important because curlews have experienced substantial population declines across much of their range with breeding populations in Great Britain and Northern Ireland having declined by over 50% during the past three decades [34], largely due to habitat loss, changes in land use and increased predation pressure [31, 35]. Similar declines have been reported across Ireland and mainland Europe [36, 37]. Although pressures during the non-breeding season may also contribute, evidence suggests that low reproductive output on the breeding sites is a key driver of population declines [38–40]. Consequently, the species is currently classified as “Near Threatened” globally and appears on the Red Lists in Great Britain and Ireland [41–43].

While previous research has described population trends and broad migratory behaviours in curlews, important gaps remain in our understanding of how breeding outcomes may influence subsequent migration behaviour. Studies of migratory birds suggest that breeding conditions and reproductive success can affect migration phenology, including the timing of departure from breeding sites ([44–47]; but see [48]). In curlews, environmental cues at breeding sites, such as vegetation green-up, have been linked to spring departure in the following year [49]. GPS-tracking studies have revealed substantial variation in reproductive outcomes among individuals, including high proportions of non-breeders and low nest survival rates [50], with successful breeding associated with later migrations in German curlew populations [51]. However, the potential role of breeding success in combination with key ecological factors such as breeding-site characteristics and sex differences, in shaping migration departure timing, distance and speed remains largely unexplored.

In this study, we investigate how the migration behaviour of curlews breeding in Great Britain is influenced by ecological and individual factors, including sex, breeding-site latitude, weather conditions, individual repeatability and reproductive success. Specifically, we examine how these factors affect migration timing, speed and distance. We predict that (1)migration behaviour differs between sexes due to variation in reproductive roles or competition (e.g., sex biased timing) [52, 53], (2) breeding latitude influences migration behaviour because of differing time constraints associated with latitude [54, 55], (3) weather conditions affect pre migration food availability and fuelling, thereby shaping migration timing, speed and distance [6–9], (4) individuals are repeatable in their migration timing across years,consistent with previous studies demonstrating high repeatability in timing, especially in spring [56, 57], and (5) reproductive success influences subsequent migration phenology and other migration parameters [44–47, 51], confounded by previously unaccounted effects of breeding latitude and sex. In addition to these main effects, we explicitly test interactions between sex and latitude, sex and weather, and sex and reproductive success, to assess whether effects differ between males and females, reflecting sex-specific constraints. To test these predictions, we used GPS-tracking data from 80 curlews breeding across a latitudinal gradient of 6.3 degrees, i.e. 703 kms, in Great Britain, allowing us to link individual and environmental predictors to specific aspects of migration behaviour.

## Methods

### Data collection

**Fig. 1:**
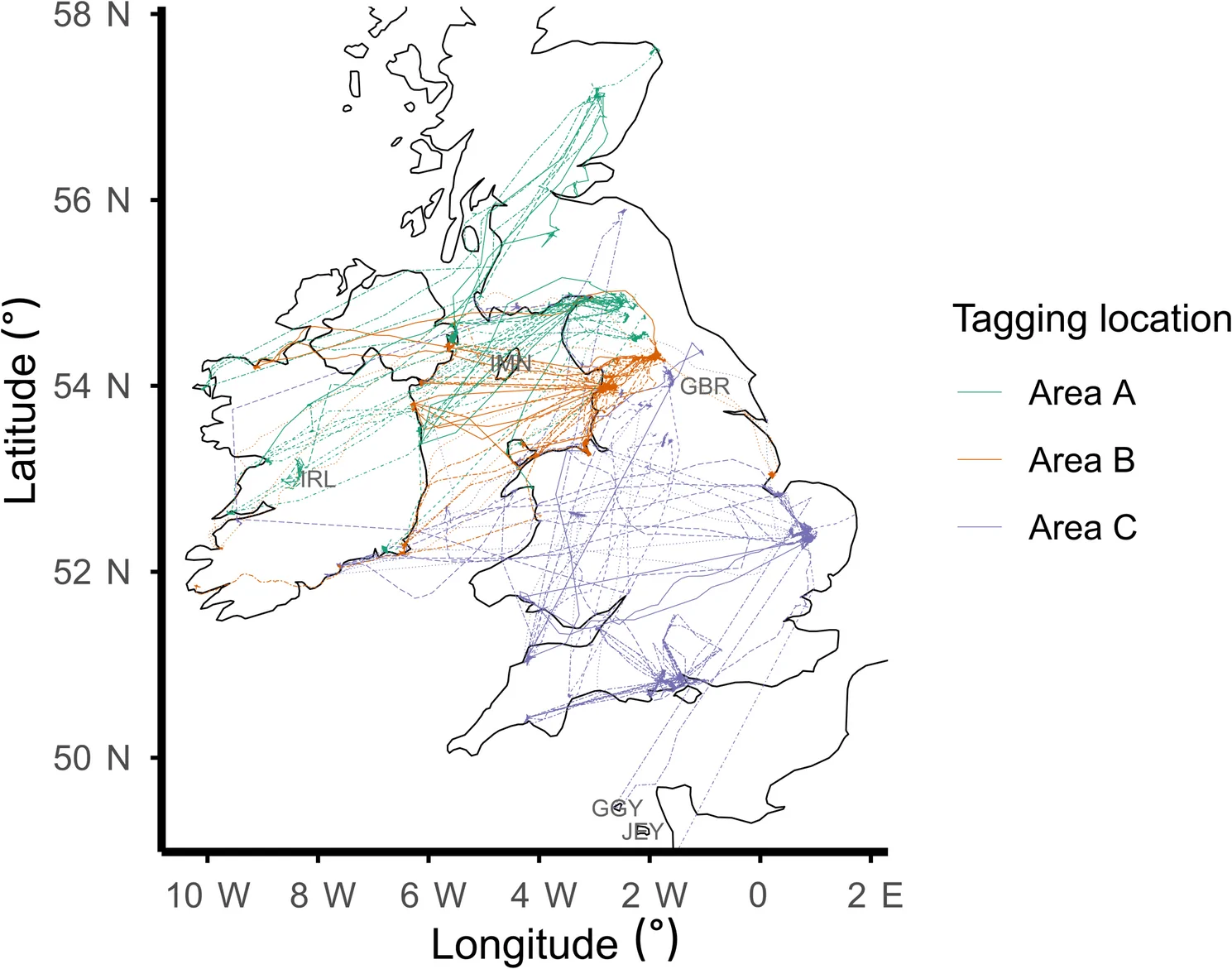
Migration tracks of Eurasian curlews breeding in Great Britain (*N* = 80). Colours represent different tagging locations encompassing Area A (*N* = 20): Aberdeenshire, Cumbria, Northumberland, County Durham, Area B (*N* = 26): Lancashire, North Yorkshire, and Area C (*N* = 34): Devon, Hampshire, Anglesey, Suffolk, Norfolk. Line types represent different individuals. Letters are country abbreviations. The track of one individual wintering in Portugal was partially removed to highlight the main migration routes.

To gather broad, representative migration data across a range of locations in Great Britain, we combined GPS-tracking datasets from several curlew studies conducted from 2018 to 2025. This provided data from 80 tagged adult curlews breeding in Britain (Table S1). Tagging occurred in 11 locations spread across Great Britain, ranging from Hampshire and Devon (min. 50.8 degrees latitude) in southern England to Aberdeenshire in Scotland (max. 57.1 degrees latitude) (Table S1, Fig. 1). Different studies employed different capture methods. During the breeding season, 68 curlews were caught between 27 March and 7 June (Table S1). About two-thirds of individuals were caught on the nest using a heart-shaped walk-in trap (ca. 1.5 m diameter) with dummy eggs, temporarily replacing the bird’s eggs. A small number of individuals were either hand-netted on the nest or caught on territory in April–early May using mist-nets with decoys and a tape-lure at night or a decoy and clap-net during the day. Twelve curlews were caught outside of the breeding season using cannon-netting or mist-netting at coastal feeding sites (between 7 November and 30 January; Table S1). All birds were fitted with a British Trust for Ornithology (BTO) metal leg ring and an individually unique combination of colour rings. Morphometric measurements, including bill length, tarsus length, wing length and mass, were recorded. We determined the sex of each bird using a published discriminant function based on bill length [58], classifying individuals as male when the estimated probability of being male was ≥ 0.5. Our dataset included 25 females and 55 males.

Curlews were fitted with a GPS-GSM tag by licensed individuals with appropriate endorsements on BTO ringing permits. The tracking devices all used GPS for location estimation and the GSM network for data transmission, but varied in mass and manufacturer between studies and over time. Specifically, we fitted six MoveTech Flyway-18 tags (18 g, 2018–2019), 15 PathTrack NanoFix tags (11 g, 2019–2022) and 59 Ornitela OrniTrack-E10 tags (13 g, 2022–2024; Table S1)

Most tags were attached using Rappole-style leg-loop harnesses [59, 60], but four birds were tagged using a temporary cyanoacrylate glue attachment on the upper back [50]. During 2018–2021, the harnesses consisted of 1.5 mm silicone cord passed through 2.0 mm ID Silastic tubing (Silastic^®^ (Dow, Midland, MI, USA)) and from 2022 they were made of 2.0 mm silicone cord (Polymax Ltd, Bordon, UK; product code: 3007102) without the tubing. The total mass added to each bird (including the tag and harness) ranged from 0.9 to 3.8% of body mass (mean 2.1% ± 0.5 SD). Mean body mass was 745 g ± 96 SD (range: 582–1097 g). On average, MoveTech tags weighed 3.2% (± 0.4 SD) of total body mass, PathTrack tags 1.9% (± 0.4 SD) and Ornitela tags 2.0% (± 0.3 SD). Except for three birds that likely reacted negatively to capture or tagging and were quickly recaptured or predated, and one bird that deserted its nest, tagging did not cause any observable adverse reactions or changes in behaviour. Tags were recharged by solar panels. Fix rates varied seasonally and between tag types. A daily mean of 179 geographic tag fixes was recorded (109.3 ± SD; range: 1-289). This equated to a fix every eight minutes on average, though this frequency varied between tags. GPS location data were remotely relayed to and stored in respective online repositories (www.pathtrack.co.uk and www.movebank.org).

## Data extraction

We imported the data from the online repositories into R (version 4.4.1; [61]) for processing and analysis. We removed duplicate fixes and unsuccessful positioning attempts. Data points with fewer than four satellite counts were excluded (5% of total data removed), as 2-D location estimation was not possible. Unlike previous fine-scale studies, which removed data with fewer than five or six satellite counts [50, 62], we retained these points as our study of migrations could tolerate slightly lower levels of precision, and doing so prevented significantly higher data loss [63–65]. Instead, we visually examined the data and removed unrealistic locations showing large distances covered over impossibly short time periods between consecutive fixes and which were outside the range of remaining points for an individual (nine positions removed). Ultimately, we obtained data from 80 individuals, yielding one or more migrations per individual across the study period (Table S1). A total of 1,760,394 fixes were included in this study.

From the raw data, we derived movement variables by calculating distances between consecutive fixes using the Vincenty ellipsoid method implemented in the *geosphere* package [51, 66]. Daily total distance travelled (km) was calculated by summing all distances within a day; thus, estimates represent the actual distance moved rather than straight-line distances between seasonal sites. Daily distance estimates were excluded only when a day contained a single fix that followed a data gap of one or more days (*N* = 69). In these cases, movement between fixes could not be reliably assigned to a specific day, precluding estimation of daily distance travelled. We retained a mean of 367 days of valid data per individual (251.6 ± SD; range: 26–942; Table S1). Finally, across each migration, daily total distance travelled was summed.

To classify annual movement phases, we used a combination of movement thresholds and seasonal context. Days with movements ≥ 40 km were classified as active migratory travel days. This threshold, although arbitrary, was chosen because curlews exhibit high site fidelity during stationary periods. Migration periods (pre-breeding and post-breeding migration) were then defined as the continuous interval encompassing these travel days as well as intervening low-movement days (< 40 km), which represent stopover behaviour. The terms “pre-breeding migration” and “post-breeding migration” refer to the migrations before and after the breeding season in spring and summer, respectively, when individuals are expected to breed, although not all necessarily do (based on field observations, 81% of individuals in this study initiated a breeding attempt in a given year). Breeding sites were assigned during the expected breeding period in spring and early summer, bounded by the end of pre-breeding migration and the onset of post-breeding migration, and characterised by spatially restricted movements. Wintering sites were assigned outside the breeding period, between post-breeding and subsequent pre-breeding migration, and were similarly characterised by limited daily movements (< 40 km). In cases where migrations were not detected by the 40 km threshold (*N* = 37 migration events), migration timing and phase assignment were determined by visual inspection of movement trajectories, identifying sustained directional displacement away from breeding or wintering areas. Departure dates were defined as the first day of migration and arrival dates as the first day at the breeding or wintering sites.

To estimate total migration duration, we explicitly incorporated the fuelling period required prior to the first migratory flight, as fuelling represents a substantial and biologically important component of overall migration time [67–70], using an adapted version of Pennycuick’s flight-range model [71] in the *afpt* package ([72]; for details on estimates of aerodynamic parameters and fuel deposition rates see Table S2). Total migration duration (d) was calculated as the sum of observed migration duration and estimated first fuelling time. In addition, migration speed was calculated as the total distance travelled during migration divided by total migration duration, yielding a mean migration speed expressed in kilometres per day (km/d). By incorporating fuelling time into the denominator, this metric reflects the realised rate of migration, including first fuelling period and stopover periods, rather than flight speed alone. Further, instantaneous ground speed estimates were not used because they are unavailable for all tag types and do not account for first fuelling time. Because aerodynamic parameters and fuel deposition rates may vary among individuals and environmental conditions, our estimates should be interpreted as approximations of fuelling requirements, migration duration and migration speed rather than exact individual predictions.

We identified nest-site locations in the field for each year and each bird where possible, accounting for 69% of breeding attempts. For the remaining 31% of breeding attempts, we estimated nest-site locations. Nesting status was confirmed for 81% of birds in a given year; for the remaining 19%, we assumed that birds occupied their breeding sites and were potential nesters, although not all may have nested. We subsetted all GPS data points recorded during the breeding season based on migration arrival and departure dates. From this subset, we applied a latitudinal and longitudinal grid (cell size 0.0001°*N* × 0.0001°W) across the curlew’s visited area to identify the probable nest site. The centroid of the grid cell with the highest count of GPS positions was designated as the nest site [49]. We assessed the accuracy of estimated nest sites by comparing them with observed nest locations for breeding attempts with field-identified locations, with a mean difference of 116 m (± 199 SD; range: 0.4–944.4 m). Using these data, we estimated nest-site fidelity across years (see Results), quantifying the distance between an individual’s nesting locations in consecutive breeding years. Nest relocation distances were calculated using great-circle (Haversine) distances based on recorded nesting latitude and longitude. For individuals with multiple breeding years, distances were computed between consecutive years only, yielding one relocation distance per inter-annual breeding interval. Because relocation distances were right-skewed, nest-site fidelity was summarised using the median and interquartile range (first quartile (Q1) - third quartile (Q3)).

We extracted key climatic variables using the European Centre for Medium-Range Weather Forecasts (ECMWF) of the Copernicus Climate Change Service. Specifically, this dataset originated from the Reanalysis 5th Generation (ERA5) hourly data, with a spatial resolution of about 31 km [73]. We extracted weather data closest to a bird’s average daily location, including hourly air temperature at 2 m above the surface (°C), total hourly precipitation (rain, snow, hail – mm), wind speed at 100 m above the surface (m/s) and wind direction at 100 m above the surface (degrees). Temperature and precipitation were averaged and summed per day, respectively, and these daily values were again averaged for temperature and summed for precipitation across the seven days preceding and including migration departure (pre- and post-breeding) to capture short-term environmental conditions that may influence food availability, fuelling rates and migration decisions (e.g [8, 74–80]). In contrast, wind speed and direction were averaged and evaluated on the day of departure only, because birds are hypothesised to respond to immediate wind conditions; tailwinds and favourable wind regimes have been shown to increase departure probabilities and reduce energetic costs during migratory flights (e.g., wind selectivity and departure behaviour in shorebirds and waterfowl) [81–84]. Wind direction was calculated as a circular mean to account for its 0°–360° nature: angles were converted to radians, averaged via sine and cosine components, and then converted back to degrees. This approach ensures that directions near 0° and 360° are treated correctly, avoiding bias that would result from a linear average.

## Statistical analyses

### Sex, latitude and weather effects on pre- and post-breeding migration

To determine whether the factors outlined in predictions 1–3, i.e. sex, breeding-site latitude and weather conditions, were associated with migration behaviour in curlews breeding in Great Britain, we constructed a series of ten models. The dependent variables were: [1] departure date [2], arrival date [3], total distance travelled during migration [4], migration duration (including first fuelling period and stopover periods) and [5] migration speed of the pre-breeding and post-breeding migration. We analysed pre-breeding and post-breeding migrations separately, constructing distinct models for each period because migration behaviours before and after the breeding season may be influenced by different selection pressures. Summary statistics for these migration variables are provided in Table 1. We performed statistical analyses using linear mixed models (LMMs) implemented in the *lme4* package [85, 86]. We fitted all LMMs with a Gaussian error distribution and identity link-function, but log-transformed distance and duration variables as the data were skewed to the right. In all models, explanatory variables of sex, breeding-site latitude, mean temperature, total precipitation, mean wind speed and mean wind direction were fitted as fixed effects. These variables were selected a priori based on the predictions that sex-specific behaviours, spatial origin and weather conditions influence the timing, distance and speed of migration. Latitude of wintering sites was not included in these analyses because we expected it to be correlated with nesting latitude, making it difficult to disentangle their effects statistically. Daily weather metrics were derived from hourly data at a bird’s average daily location and aggregated across the period leading up to migration departure (see above). Interactions between the explanatory variables and sex were tested for but removed from the model if not significant. We also fitted year as a categorical variable to control for inter-annual variation. We accounted for repeated measures by incorporating individual identity as a random intercept.

**Table 1:** Summary statistics of the migration parameters departure date, arrival date, total distance travelled (km), duration (d) and speed (km/d) during the (a) pre-breeding period and (b) post-breeding period. Provided are sample sizes (N), mean, median, minimum (min), maximum (max), standard deviation (SD), first quartile (Q1) and third quartile (Q3). Months of the year are abbreviated

Period	Parameter	*N*	Mean	Median	Min	Max	SD	Q1	Q3
(a) Pre-breeding	Dep. dates	63	27 Feb	27 Feb	3 Feb	30 Mar	11.3	18 Feb	8 Mar
	Arr. dates	63	5 Mar	5 Mar	6 Feb	24 Apr	14.1	21 Feb	13 Mar
	Distance	63	287	238	7	980	250.5	60.8	421.0
	Duration	63	14	11	2	50	11.4	4.5	18.5
	Speed	63	19	23	3	33	8.5	12.3	25.5
(b) Post-breeding	Dep. dates	117	24 Jun	24 Jun	27 Apr	2 Aug	16.8	12 May	4 Jul
	Arr. dates	117	1 Jul	30 Jun	29 Apr	23 Oct	26.3	15 Jun	9 Jul
	Distance	117	267	213	9	1120	253.4	56.5	410.7
	Duration	117	16	9	2	172	24.1	3.8	16.5
	Speed	117	19	21	2	32	7.5	12.9	24.9

## Individual repeatability of migration timing

To quantify the consistency of individual pre-breeding and post-breeding departure dates (prediction 4), we calculated repeatability using LMMs [87]. Departure date was modelled as the response variable with individual identity included as a random effect. To account for interannual variation in migration phenology, year was included as a fixed effect. Repeatability (R) was estimated from the variance attributable to individual differences relative to total variance using the *rptR* package [88]. We specified a Gaussian error distribution. Statistical support for repeatability was assessed using both parametric bootstrapping (1,000 iterations) to obtain 95% confidence intervals and permutation tests (1,000 iterations) to evaluate significance.

### Effects of breeding success on post-breeding migration

Finally, to investigate whether breeding success influenced consequent migration behaviours in a given year (prediction 5), we conducted analyses on a subset of the data (*N* = 91 breeding attempts) for which field observations of individual birds informed by GPS locations and identification from colour rings enabled classification of breeding attempts as successful guarding of at least one chick beyond 30 days (minimum age at fledging [89]), or failure during incubation or chick rearing prior to 30 days. For some birds (e.g. from North Yorkshire), we used methodology developed by Bowgen et al. [50] to confirm chick guarding alongside field observations. We first analysed breeding success (successful versus failed) using a generalized linear mixed model (GLMM) model with a binomial error distribution and a logit link function, implemented in the *lme4* package [85, 86]. GLMMs were checked for overdispersion by calculating the ratio of the sum of squared Pearson residuals to the residual degrees of freedom, following standard practice for binomial GLMMs [90]. Values below one indicate slight under-dispersion; no evidence of problematic overdispersion was found (observed value = 0.29). Latitude was included as a fixed variable to test for the effect of nest location (north versus south) on breeding success. Individual identity was included as a random intercept to account for repeated observations of the same individual. Second, we ran the same five model structures as described above on the post-breeding migration variables, including breeding success of the current year in addition to previously identified significant covariates. Breeding success was placed in interactions with sex and with latitude; these interactions were dropped from the model if not significant. As before, we accounted for repeated measures by including individual identity as a random intercept.

All continuous, independent fixed variables were standardised to a mean of zero and a standard deviation of one to allow for comparable estimates across fixed effects within models [91]. To assess multicollinearity, we calculated variance inflation factors (VIFs) using the “VIF” function in the *car* package [92], confirming that multicollinearity was minimal across all our models (all generalised VIFs^(1/(2*Df)) < 2.1; [93, 94]. Dependent variables were visually inspected for normality using histograms [95]. Variables with right-skewed distributions, i.e. distance and duration, were log-transformed prior to modelling. Full linear mixed models were checked for normally distributed and homogenous residuals through QQ-plots and residuals versus fitted values [62, 63]. All models indicated acceptable adherence to model assumptions. Outliers were examined across datasets and a single bird, that made an exceptional 1,901 km post-breeding migration to overwinter in Portugal, was excluded from all formal migration analyses.

To assess model stability, we applied the approach of Nieuwenhuis et al. [96]. Each model was refitted while systematically excluding one level of the random effects at a time, and the resulting fixed effect estimates were compared to those from the full model to identify any influential groups. Results showed high model stability, with no significant influence from any specific level of the random effects (i.e., no influential individual curlew identities). Finally, we calculated effect sizes as the proportion of variance explained by fixed effects only (marginal R^2^_m_) and by both fixed and random effects (conditional R^2^_c_) [97] using the “r.squared GLMM” function in *MuMIn* package [98]. We report effect sizes, standard errors, t or z values, degrees of freedom (DF), chi-squared values and the significance of each variable, with a significance threshold set at *P* ≤ 0.05, by evaluating changes in deviance between full models and models with individual terms excluded using the *ANOVA* function and a backward elimination procedure. We also fitted several specific interactions (see below). However, these interactions were removed if they failed to have significant explanatory power based on changes in deviance in a first step before removing non-significant single variables.

## Results

**Fig. 2:**
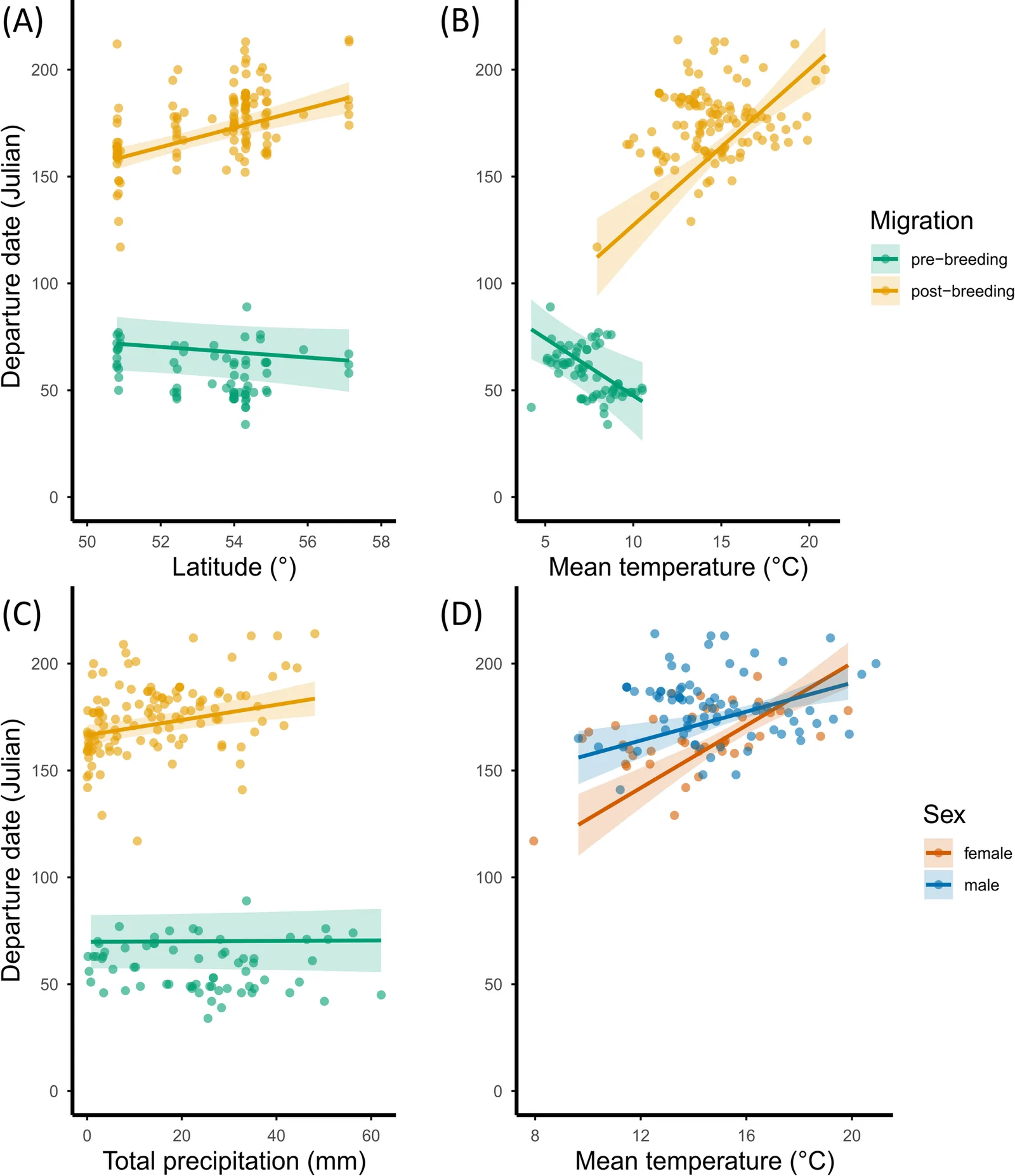
Pre- and post-breeding migration departure dates (Julian units with 1 = 1 January) in relation to breeding-site latitude (°) (**A**), mean temperature (°C) (**B**), total precipitation (mm) (**C**) and an interaction between mean temperature and sex in autumn (**D**). Represented are the predicted lines of best fit and 95% confidence intervals estimated from the models (Table S3 and S4) and raw data points

Based on descriptive summaries of tracking data (Fig. 1), tagged curlews breeding farther north (*N* = 46; Scotland and northern England (Area A and B) in Fig. 1) predominantly migrated southwest to west, with 58% wintering in Ireland. The remainder of tagged curlews migrated to the western British coastline and estuaries such as the Solway Firth and Dee Estuary. Two individuals migrated to the east, close to the Buchan and Firth of Forth coastlines in Scotland. In contrast, curlews breeding in southern Great Britain (*N* = 34; southern England in Fig. 2) exhibited greater variation in migration destinations, with only 9% wintering in Ireland. Most southern breeders migrated to the British coastline (e.g. the Exe Estuary, Poole Harbour and Solent), although two individuals wintered as far south as Guernsey and Portugal.

## Nest site fidelity

Individuals breeding across multiple years showed high nest-site fidelity.

Nest relocation distances were right-skewed, with a median of 0 m (Q1-Q3: 0–49 m) for all individuals (*N* = 41), indicating that many individuals reuse the same nesting location across years. Females exhibited larger nest relocation distances than males (female median: 0, Q1-Q3: 0–89 m (*N* = 10); male median: 0, Q1-Q3: 0–22 m (*N* = 31)).

### Sex, latitude and weather effects on pre-breeding migration

We tested whether sex, breeding-site latitude and local weather conditions (predictions 1–3) were related to pre-breeding migration departure timing in 80 curlews breeding across a 703-km north–south gradient in Great Britain (Fig. 1). Consistent with our prediction, male curlews departed on average seven days earlier than females (*χ²* = 5.47, *P* = 0.019). However, departure dates did not differ with latitude (mean = 27 February ± 11.3 SD; *χ²* = 1.97, *P* = 0.160; Fig. 2A; Table 1). Departure timing was related to local weather conditions experienced in the week leading up to migration. Earlier departures were significantly correlated with warmer conditions, with a 1 °C increase in temperature leading to ca. five days advancement in migration departure (*χ²* = 6.089, *P* = 0.025; Figs. 2B). Total precipitation, mean wind speed and mean wind direction were not correlated with departure timing (Table S3). Departure timing varied significantly between years, with curlews departing particularly early in 2021 compared to the other years (*χ²* = 34.39, *P* < 0.001). We found no significant interactions of sex-latitude or sex-weather variables preceding breeding (Table S3).

Arrival dates were strongly correlated with departure dates during pre-breeding migration (Pearson’s correlation: r [65] = 0.88, *P* < 0.001). Mean arrival date at breeding sites was 5 March (± 14.1 SD; Table 1). Arrival at breeding sites did not differ between sexes (*χ²* = 2.34, *P* = 0.126). As for departure dates, northern breeders arrived at similar times at breeding sites as southern breeders (*χ²* = 0.44, *P* = 0.505). Warmer conditions experienced in the week leading up to migration were significantly correlated with earlier arrivals, with an advancement of ca. eight days per 1 °C increase in mean temperature (*χ²* = 10.57, *P* = 0.001). Total precipitation, wind speed and wind direction were not correlated with arrival timing (Table S2). Arrival timing varied significantly between years, with 2022 and 2024 having comparably earlier arrivals than the other study years (*χ²* = 12.84, *P* = 0.025). We did not find significant effects of sex–latitude or sex–weather interactions on arrival timing (Tables S3).

Curlews travelled a total of 238 km during pre-breeding migration (median, Q1-Q3: 61–421 km; Table 1). We found no differences in migration distance between sexes (*χ²* = 0.70, *P* = 0.402). Individuals breeding in Scotland and northern England migrated longer distances than those breeding in southern England, with an observed 51% increase in distance per degree in latitude (*χ²* = 10.89, *P* < 0.001). Wind direction significantly affected total distance travelled during migration in a sex-specific manner (*χ²* = 7.98, *P* = 0.005). In females, a one standard deviation (73°) clockwise shift in wind direction was associated with a ca. 13% reduction in migration distance. During spring migration, the average wind on the day of departure was 229° (southwest), while the average migration direction of birds was 60° (east-northeast), meaning that this shift corresponds roughly to winds rotating toward northwest. In contrast, males showed no detectable response to wind direction. There were no differences in migration distance between sexes breeding at different latitudes, nor were there any significant effects of the remaining weather variables as single effects or in interactions with sex (Table S3). Finally, there was significant annual variation in distance travelled during pre-breeding migration, with particularly large distances travelled in 2021 compared to the other years (*χ²* = 529.41, *P* < 0.001).

Including first fuelling period and stopover periods, we found that curlews migrated for a total of 11 days preceding breeding (median, Q1-Q3: 4.5–18.5 days; Table 1). We found no sex effect (*χ²* = 0.062, *P* = 0.803). There was a significant effect of nesting latitude on migration duration with a 29% increase in duration per degree of latitude (*χ²* = 11.49, *P* < 0.001). None of the weather and interactive variables explained significant variation in migration duration (Table S3). Year was a significant predictor: in particular, migration durations were shorter in 2021 than in the other years (*χ²* = 96.39, *P* < 0.001).

We recorded a migration speed of 19 km/d preceding breeding (= median ± 8.5 SD; Table 1). We again found no sex effect (*χ²* = 1.61, *P* = 0.205). Birds breeding at more northern latitudes migrated at higher speeds; for each degree increase in latitude, birds flew ca. 2.2 km/d faster (*χ²* = 9.44, *P* = 0.002). Birds also migrated more quickly when they experienced warmer temperatures in the lead up to spring migration; for each degree increase in mean temperature experienced in the week leading up to migration, curlews flew 1.9 km/d faster from wintering to breeding sites (*χ²* = 7.45, *P* = 0.006). All weather and interactive effects were non-significant (Table S3). There was annual variation in migration speeds, with individuals flying relatively faster in 2023 and 2024 than in the remaining years (*χ²* = 27.98, *P* < 0.001).

### Sex, latitude and weather effects on post-breeding migration

Mean departure date of curlews from their breeding sites was 24 June (± 16.8 SD; Table 1). A significant interaction between sex and temperature was detected (χ² = 7.15, P = 0.007; Fig. 2B and D); at lower mean temperatures in the week leading up to migration, females departed earlier than males, though with warmer mean temperatures, departures between the sexes did not differ. Wetter conditions preceding migration were correlated with later departures (χ² = 12.61, P < 0.001; Fig. 2C), with no significant differences between the sexes (Table S4). Northern breeders departed significantly later on post-breeding migration compared to those breeding farther south, ca. five days later for each degree increase in latitude (*χ²* = 38.84, *P* < 0.001; Fig. 2A). Further, wind direction on the day of departure significantly influenced autumn migration timing (*χ²* = 5.22, *P* = 0.022). A 90° clockwise shift in wind direction was associated with ca. 2.6 days later departure. The average wind on the day of departure was 237° (west-southwest), while the average migration direction of birds was 245° (west-southwest), meaning that this shift corresponds roughly to winds rotating toward north-northwest. We found no significant effects of year and wind speed, nor of interactions between sex and latitude or sex and other weather variables (Table S4).

Following breeding, arrival dates were also strongly correlated with departure dates (r [118] = 0.63, *P* < 0.001). The mean arrival date was 1 July (± 26.3 SD; Table 1). There was a trend for males to arrive later at wintering sites than females (*χ²* = 3.70, *P* = 0.054). Northern breeders arrived significantly later than southern breeders; for each degree increase in latitude birds arrived ca. three days later (χ² = 7.36, *P* = 0.007). Weather preceding and during migration influenced arrival timing: warmer, wetter and calmer conditions experienced during the week leading up to migration resulted in later arrivals at wintering sites (mean temperature: *χ²* = 8.38, *P* = 0.004; total precipitation: *χ²* = 20.92, *P* < 0.001, mean wind speed: *χ²* = 5.65, *P* = 0.017). We did not find significant effects of year, wind direction and sex-latitude or sex-weather interactions on arrival timing (Tables S4).

Curlews travelled a total of 213 km following breeding (median, Q1-Q3: 57–411 km; Table 1). There were no differences in migration distance between sexes (*χ²* = 0.68, *P* = 0.409). Individuals breeding in Scotland and northern England migrated longer distances than those breeding in southern England, with an observed 42% increase in distance per degree in latitude (*χ²* = 25.28, *P* < 0.001). There were no significant weather, interactive or annual effects on migration distance (Table S4).

Curlews migrated for nine days in total (median including first fuelling period and stopover periods; Q1-Q3: 3.8–16.5 days; Table 1). There were no significant differences in migration duration between sexes (*χ²* = 0.079, *P* = 0.779). Birds breeding at more northern latitudes migrated for longer durations, by ca. 26% per degree of latitude (*χ²* = 16.89, *P* < 0.001). We found no other significant effects on migration duration (Table S4).

We recorded that curlews migrated at a speed of 19 km/d following breeding (median ± 7.5 SD; Table 1). Females flew ca. 4.9 km/d faster than males (*χ²* = 5.45, *P* = 0.020). Birds breeding at more northern latitudes migrated at higher speeds, ca. 2.5 km/d faster per degree of latitude (*χ²* = 24.96, *P* < 0.001). Drier weather conditions experienced during the week leading up to migration increased migration speed (*χ²* = 4.46, *P* = 0.035; Table S4). Specifically, each 1 mm reduction in precipitation was associated with a ca. 0.15 km/d increase in migration speed. All other effects were non-significant as single effects and within interactions (Table S4).

### Individual repeatability of migration timing

Departure timing showed relatively high repeatability within individuals in both migration periods (prediction 4). Pre-breeding departure dates suggest relatively high repeatability (*R* = 0.55, 95% CI: 0.14–0.81; *N* = 69 observations, 53 individuals), with repeatability significantly greater than zero based on both likelihood ratio (*χ²* = 4.77, df = 1, *P* = 0.014) and permutation tests (*P* = 0.014). Similarly, post-breeding departure dates showed high repeatability (*R* = 0.59, 95% CI: 0.37–0.75; *N* = 120 observations, 75 individuals) and were strongly supported by both likelihood ratio (*χ²* = 25, df = 1, *P* < 0.001) and permutation tests (*P* = 0.001), indicating consistent individual differences in departure timing across seasons.

### Effects of breeding success on post-breeding migration

**Fig. 3:**
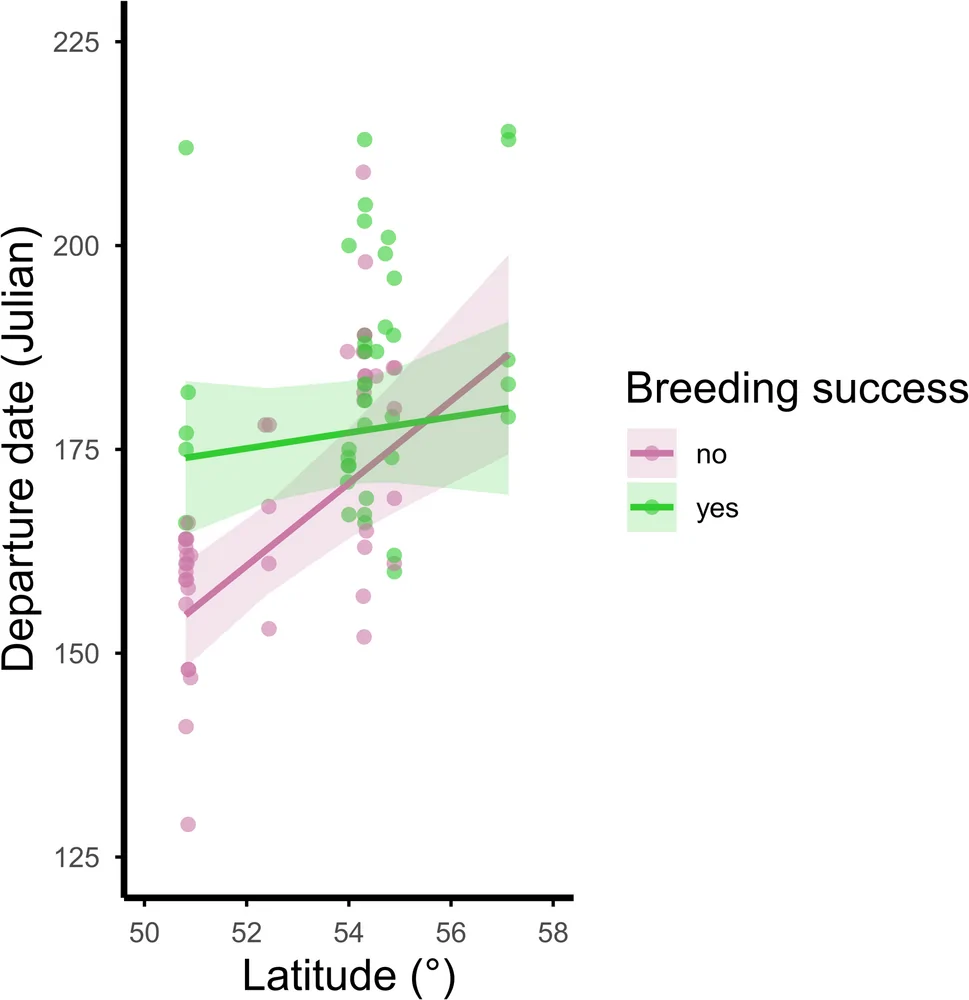
Post-breeding migration departure dates in relation to breeding success (yes = parents successfully guarded at least one chick beyond 30 days (*N* = 41), no = nesting attempt failed during incubation or rearing prior to this threshold (*N *= 50)) and breeding-site latitude. Represented are the predicted lines of best fit and 95% confidence intervals estimated from the models (Table S5) and raw data points

In a subset of the data where breeding outcome was known, we investigated whether breeding success affected post-breeding migration (prediction 5). We found that 41 breeding attempts were successful, while 50 nests or broods failed prior to fledging. Departure dates were significantly influenced by a breeding success - latitude interaction: successful breeders departed on average eight days later on post-breeding migration than failed breeders, and this effect was mainly driven by southern compared to northern breeders (*χ²* = 8.16, *P* = 0.004, Fig. 3). Post-hoc analysis revealed that the effect of successful breeding correlating with post-breeding departure was present in both females (*χ²* = 9.58, *P* < 0.002) and males (*χ²* = 5.67, *P* = 0.017). There was no interactive effect of breeding success and sex on departure dates (*χ²* = 1.51, *P* = 0.218). We found no significant single or interactive effects of breeding success on post-breeding migration arrival dates, distance, duration or speed (Table S5).

## Discussion

Our study revealed spatial and temporal variation in migrations of curlew populations breeding in Great Britain correlated with both breeding origin and individual-level factors. Migration behaviours varied with sex, breeding-site latitude, local weather conditions, sex (predictions 1–3) and between individuals (prediction 4). Males departed earlier on migration preceding breeding and later following breeding, but migrated faster to wintering sites than females. Northern breeders migrated later following breeding, migrated farther and for longer despite travelling faster than southern breeders. Additionally, local weather, particularly temperature and precipitation, significantly influenced departure and arrival timing and migration speed. Finally, failed breeding was associated with earlier departure during post-breeding migration (prediction 5), an effect driven mainly by southern breeders but not breeders in the north, where breeding success was generally higher. These results highlight the complex interplay of geographic, climatic and individual factors shaping curlew migration.

Our results revealed sex-specific responses reflecting contrasting pressures on migration timing. Females were more sensitive to temperature and adjusted pre-migration distances in response to unfavourable winds, consistent with broader research showing wind influences movement [99, 100]. Their stronger response may reflect higher wing loading, increasing reliance on wind assistance. Males departed earlier from wintering sites, likely to secure breeding territories, which may limit their ability to wait for favourable winds [4, 101]. Sex-specific feeding constraints may also play a role: males’ shorter bills limit access to deep-burrowing prey in cold conditions and instead favour the use of more readily available earthworms in grasslands [58, 102], while females adjust migration to optimise efficiency. Despite these differences, sex did not affect overall migration distance or duration, though males departed earlier before and later after breeding. This pattern aligns with Kämpfer et al. [51], who reported earlier post-breeding departure in females and failed breeders and found no sex differences in migration duration. Similar to previous studies in curlews [103, 104], we also observed a non-significant trend for females to arrive earlier at wintering sites. Earlier migration in females is likely linked to differences in parental roles, as males assume sole responsibility for chick care during the latter part of the rearing period [53]. Furthermore, Females migrated faster post-breeding, likely balancing selection to optimise trade-offs among speed, stopover refuelling and predation risk to arrive earlier at wintering sites with improved survival prospects [105–107]. Overall, these results indicate that male and female curlews differ in their migration behaviours and responses to environmental conditions, with shifting costs and benefits shaping optimal migration behaviours [3–5]. Further work is needed to identify precise drivers of these behaviours, such as habitat use and availability across wintering and breeding sites.

We found spatial structuring of migration in curlews breeding across a 703-km north–south gradient across Great Britain. Consistent with continental European populations, more northerly breeders undertook longer migration distances in both seasons and exhibited chain migration, whereby the latitudinal ordering of breeding sites was maintained on the wintering sites [29]. This latitudinal structuring is consistent with ring-recovery and GPS studies of British breeders [26–28], which show northern birds primarily wintering farther south-west in Ireland, while southern breeders winter farther south with greater variation in wintering locations (see Fig. 1). Notably, the only two individuals wintering in continental Europe (Guernsey and Portugal) were tagged in south-eastern Great Britain. In addition to latitudinal structuring, we found evidence for individual-level structuring of migration. Departure timing was significantly repeatable, indicating consistent inter-individual differences and thus stable individual schedules, and this pattern matches findings from continental curlew populations and other *Numeniini* species [51, 108, 109]. Interestingly, repeatability was similar between pre- and post-breeding departures (*R* = 0.55 versus *R* = 59, respectively), which goes against previous findings of lower repeatability in departures from breeding sites than wintering sites [57, 110]. This indicates that individual curlews show stable behavioural differences in migration timing across years and seasons. High repeatability of both autumn and spring timing has been documented in other migrants (e.g., seabirds and large raptors), suggesting that strong endogenous control of migratory schedules can produce consistent phenology in both seasons [111, 112]. Repeatability in departure timing, together with strong nest-site fidelity among individuals tracked over multiple years, further supports the presence of spatial structuring within the population.

Northern birds departed later after breeding and migrated longer distances, although they travelled at higher speeds than southern birds. These results are consistent with a meta-analysis of 27 migratory bird species showing that migration speed increases with migration distance and that departure timing is linked to arrival timing [113]. Nevertheless, this meta-analysis did not account for the initial migratory fuelling period, which may have led to an overestimation of migration speed and an underestimation of total migration duration. In contrast to continental European populations [29, 49, 103], northern breeders in our study spent a longer, rather than shorter, period on the breeding sites than southern breeders. Although this may appear unexpected given shorter reproductive windows at higher latitudes due to delayed vegetation green-up and prey availability [49], British breeders migrated relatively shorter distances than continental populations [29, 49, 51, 103], suggesting that other ecological factors may influence this pattern (e.g., food availability and migration fuelling requirements, territory quality, population density and predator pressure). Together, these findings highlight the strong influence of breeding latitude on migration behaviour and suggest behavioural decisions that maximise time spent on breeding sites, although further work is needed to identify the ecological drivers underlying these differences.

We did not observe clear seasonal differences in migration duration and speed in British breeders (median duration: 11 days preceding breeding vs. nine days following breeding; mean speed: 19 km/d in both seasons; Table 1). This contrasts with the common expectation that birds migrate faster in spring than autumn due to stronger time-selection pressures associated with early arrival at breeding sites and increased reproductive success, a pattern supported by several meta-analyses [114, 115]. However, exceptions occur across taxa [67, 116, 117]. For example, in the closely related long-distance migratory *Numenius phaeopus islandicus*, migration duration is shorter and speed is higher in autumn than in spring [67]. As in our study, that analysis incorporated the fuelling period preceding the first migratory flight, which represents a substantial component of total migration time [68–70] and may explain deviations from previous findings. Additionally, because British-breeding curlews undertake relatively short migrations compared with continental populations, there may be little advantage in prolonging post-breeding migration, resulting in similar migration durations across seasons. Continental breeders, by contrast, may benefit from dividing their longer post-breeding migrations into multiple stages interspersed with stopovers [29]. Together, these results contribute to a growing body of evidence demonstrating substantial heterogeneity in migratory behaviours among populations of migratory birds [2, 118–120].

Local weather significantly influenced migration parameters. For instance, warmer conditions, specifically higher mean temperatures during the seven days leading up to migration, were associated with earlier pre-breeding departures from wintering sites and arrivals at breeding sites, as well as higher migration speeds. Consistent with this, a small study of curlews wintering in the Dutch Wadden Sea suggested behavioural flexibility, with birds moving further afield during cold spells [121]. Our results support our expectation that weather conditions before and during migration are closely linked in this population, where migrations occur over relatively small geographic scales. In contrast, Schwemmer et al. [103] found no weather effects on departure from wintering sites in German curlews that migrate longer distances and may therefore be less flexible in adjusting to short-term changes in weather conditions.

Breeding outcomes were strongly associated with migration timing. Curlews that failed to raise chicks departed earlier on migration, especially southern breeders, and this occurred in both sexes despite males’ extended post-hatching care [53]. Early breeding failure, while females still provide care, appears to advance migration, consistent with studies linking reproductive outcomes and breeding phenology to migration schedules (e.g [122, 123]). Post-hoc analyses confirmed that hatching success was associated with later departures, with the effect again being stronger in more southern breeders (hatching success - latitude interaction: *χ²* = 4.86, *P* = 0.043). Consequently, differences in departure timing between successful and unsuccessful breeders were relatively more pronounced at southern than northern sites. These findings are consistent with previous observations in German curlews that successful breeders remain at breeding sites longer than unsuccessful pairs [51], while also revealing previously undocumented latitudinal structuring in this relationship. In contrast to previous findings in continental breeders [29], northern breeders’ longer migrations may necessitate extended breeding-site stays to refuel regardless of breeding success, whereas southern breeders leave sooner, possibly due to less favourable post-breeding conditions. Early departure may also reflect the release of parental care constraints, allowing access to safer or higher-quality roosting habitats [124]. This effect is especially relevant at southern sites with nearby estuarine resources, such as the Solent near the New Forest, which may support earlier post-failure movements from local breeding habitats that cannot fully support adult energetic requirements [125].

The higher breeding success observed in northern Great Britain may reflect more favourable land management practices for curlews [31, 126]. Curlews preferentially breed in specific grassland and wetland habitats, particularly unimproved upland grasslands and wetter mire systems, which are associated with higher reproductive success [62, 127, 128]. Across Great Britain, habitat degradation resulting from agricultural intensification, landscape fragmentation and woodland expansion has been linked to substantial declines in wader populations, including curlews [31, 127, 129–131]. In contrast, in the north of Great Britain, large areas of moorland and rough grassland are intensively managed for red grouse *Lagopus scotica* involving intensive landscape-scale predator control, which has been linked to higher curlew abundance and productivity than moorland not managed for grouse [126, 132]. The lower breeding success observed in southern breeders, together with their earlier departures and shorter residence times at breeding sites, may reflect higher rates of predation, possibly combined with less suitable habitat conditions. Additionally, earlier mowing in southern latitudes compared to northern latitudes may lead to greater egg and chick losses [133]. Hence, local management likely contributes to the latitudinal results in breeding success observed in our study.

## Conclusions

Our study revealed variable but repeatable migratory behaviours in curlews with clear spatial structuring, although these patterns were influenced by breeding success. This behavioural plasticity suggests that individuals adjust migration timing according to breeding outcomes, with unsuccessful birds departing earlier while successful pairs remain longer at breeding sites to maximise reproductive investment. Such migration behaviours are likely to be closely linked to latitudinal differences in habitat and land management. Declining breeding productivity may therefore contribute to earlier arrival and prolonged use of coastal wintering sites, particularly among southern populations. Although low productivity is driving current declines, population modelling indicates that adult survival remains the most influential factor shaping long-term population dynamics in this delayed-maturity, long-lived species. Consequently, ensuring favourable conditions that maximise adult survival, particularly in the context of earlier arrival at post-breeding and wintering sites, is critical to prevent further population declines [39, 40, 134–136]. Regional declines and range contraction in southern populations may be further exacerbated by climate change, as warming conditions are expected to reduce the relative suitability of more southerly breeding areas [137]. The interaction between climate-driven redistribution and existing spatial variation in productivity and migration behaviour could therefore reinforce current population trends and increase reliance on northern breeding and wintering habitats, suggesting that these areas are likely to become increasingly important targets for conservation action. Effective conservation requires a landscape-scale approach that prioritises habitat quality during the breeding season to boost recruitment, protects key breeding, migratory and wintering sites and mitigates threats such as nest and chick predation to ensure long-term population viability of curlew and other wader populations.

## Supplementary Information

Below is the link to the electronic supplementary material.

Supplementary Material 1

## Data Availability

The dataset generated and analysed during the current study is publicly available in the Zenodo repository at https://doi.org/10.5281/zenodo.21452573.
