# Supplementary material for: Latitudinal variation in seasonal migration of Eurasian curlews Numenius a. arquata breeding in Great Britain: breeding failure advances departure timing in southern populations
Source: Mov Ecol. 2026 Jul 23;14:65. doi: 10.1186/s40462-026-00683-5 (PMC13576177; doi:10.1186/s40462-026-00683-5)
Supplement: Supplementary file 1 [file 40462_2026_683_MOESM1_ESM.docx]

**Electronic supplementary material for Bründl et al. 2025 “****Latitudinal variation in seasonal migration of Eurasian curlews *Numenius a. arquata* breeding in Great Britain: breeding failure advances departure timing in southern populations”**

Authors: Aisha C. Bründl, Christopher J. Heward, Samantha E. Franks, Ryan A. Burrell, Harry Ewing, Eleanor M. Rivers, David Scott, Peter M. Potts, Katharine M. Bowgen, Andrew N. Hoodless

Journal: Movement Ecology

Table S1: Details of tagged curlews from 2018-2024 with associated tagging region, UK (N = 80).

| Tag type | Tag ID | Sex | Tag region | Start (day of tagging) | End (last day of data) | N days of valid data |
| --- | --- | --- | --- | --- | --- | --- |
| Movetech | 886 | M | Hampshire | 07/06/2018 | 07/07/2018 | 30 |
| Movetech | 887 | F | Hampshire | 25/05/2018 | 29/06/2018 | 35 |
| Movetech | 892 | M | Hampshire | 20/05/2019 | 21/09/2019 | 125 |
| Movetech | 904 | M | Hampshire | 25/05/2018 | 19/06/2018 | 26 |
| Movetech | 907 | M | Aberdeenshire | 22/05/2018 | 03/09/2018 | 92 |
| Movetech | 1103 | F | Hampshire | 30/05/2019 | 20/09/2022 | 603 |
| Ornitela | 220566 | M | Cumbria | 18/05/2022 | 05/04/2024 | 689 |
| Ornitela | 220567 | M | Lancashire | 22/05/2022 | 10/09/2024 | 843 |
| Ornitela | 220568 | F | Hampshire | 30/05/2022 | 11/09/2024 | 806 |
| Ornitela | 220569 | F | Northumberland | 21/05/2022 | 16/12/2022 | 67 |
| Ornitela | 220570 | M | Hampshire | 29/05/2022 | 04/09/2024 | 816 |
| Ornitela | 220571 | M | Northumberland | 21/05/2022 | 08/08/2022 | 78 |
| Ornitela | 220894 | M | Norfolk | 02/04/2022 | 27/05/2023 | 211 |
| Ornitela | 220895 | F | Norfolk | 02/04/2022 | 19/07/2023 | 404 |
| Ornitela | 220897 | M | Suffolk | 02/04/2022 | 22/06/2023 | 266 |
| Ornitela | 220898 | F | Suffolk | 02/04/2022 | 15/10/2023 | 347 |
| Ornitela | 220901 | M | Norfolk | 02/04/2022 | 20/07/2022 | 90 |
| Ornitela | 220902 | M | Suffolk | 08/05/2022 | 12/01/2025 | 942 |
| Ornitela | 220903 | M | Norfolk | 02/04/2022 | 17/02/2023 | 232 |
| Ornitela | 220905 | M | Norfolk | 02/04/2022 | 10/01/2025 | 882 |
| Ornitela | 220906 | F | Norfolk | 02/04/2022 | 07/10/2022 | 188 |
| Ornitela | 220915 | M | Northumberland | 16/05/2022 | 29/12/2022 | 228 |
| Ornitela | 220916 | F | Hampshire | 01/05/2022 | 11/09/2024 | 857 |
| Ornitela | 220917 | M | Cumbria | 21/05/2022 | 28/09/2022 | 131 |
| Ornitela | 220918 | M | Lancashire | 20/05/2022 | 11/09/2024 | 832 |
| Ornitela | 220919 | M | Northumberland | 17/05/2022 | 27/08/2023 | 468 |
| Ornitela | 220920 | F | Hampshire | 01/05/2022 | 10/09/2024 | 862 |
| Ornitela | 220921 | M | Northumberland | 18/05/2022 | 10/12/2022 | 115 |
| Ornitela | 220922 | M | Northumberland | 21/05/2022 | 01/07/2023 | 407 |
| Ornitela | 220923 | F | Northumberland | 17/05/2022 | 13/10/2022 | 139 |
| Ornitela | 220924 | M | Northumberland | 18/05/2022 | 12/03/2024 | 665 |
| Ornitela | 223550 | M | Lancashire | 06/06/2022 | 17/02/2023 | 257 |
| Ornitela | 223552 | M | Lancashire | 18/05/2023 | 11/09/2024 | 483 |
| Ornitela | 223555 | F | Lancashire | 06/06/2022 | 11/09/2024 | 829 |
| Ornitela | 225147 | F | North Yorkshire | 11/04/2023 | 19/09/2024 | 447 |
| Ornitela | 225148 | M | North Yorkshire | 13/04/2023 | 22/05/2024 | 406 |
| Ornitela | 225150 | M | North Yorkshire | 13/04/2023 | 23/08/2024 | 492 |
| Ornitela | 225151 | M | North Yorkshire | 16/04/2023 | 29/03/2024 | 348 |
| Ornitela | 225152 | M | North Yorkshire | 17/04/2023 | 16/10/2024 | 545 |
| Ornitela | 225153 | M | North Yorkshire | 18/04/2023 | 15/10/2024 | 520 |
| Ornitela | 225154 | M | North Yorkshire | 18/04/2023 | 29/08/2023 | 134 |
| Ornitela | 225155 | M | North Yorkshire | 19/04/2023 | 15/10/2024 | 546 |
| Ornitela | 225156 | F | North Yorkshire | 19/04/2023 | 30/07/2023 | 103 |
| Ornitela | 225157 | M | North Yorkshire | 22/04/2023 | 22/10/2024 | 542 |
| Ornitela | 225158 | F | North Yorkshire | 14/04/2023 | 21/01/2024 | 275 |
| Ornitela | 225159 | M | North Yorkshire | 26/04/2023 | 23/09/2024 | 494 |
| Ornitela | 225160 | M | North Yorkshire | 23/04/2023 | 15/10/2023 | 176 |
| Ornitela | 225161 | M | North Yorkshire | 24/04/2023 | 14/11/2024 | 551 |
| Ornitela | 225162 | M | North Yorkshire | 25/04/2023 | 01/11/2024 | 523 |
| Ornitela | 225163 | M | North Yorkshire | 27/04/2023 | 30/12/2024 | 586 |
| Ornitela | 225164 | F | North Yorkshire | 24/04/2024 | 14/11/2024 | 200 |
| Ornitela | 225165 | M | North Yorkshire | 28/04/2023 | 26/09/2024 | 491 |
| Ornitela | 226217 | M | Devon | 15/12/2023 | 12/09/2024 | 272 |
| Ornitela | 226218 | F | Devon | 15/12/2023 | 12/09/2024 | 272 |
| Ornitela | 231631 | M | Lancashire | 15/05/2023 | 01/01/2024 | 231 |
| Ornitela | 231632 | M | County Durham | 20/05/2023 | 24/05/2024 | 371 |
| Ornitela | 231633 | M | County Durham | 18/05/2023 | 27/09/2023 | 133 |
| Ornitela | 231634 | M | Northumberland | 21/05/2023 | 12/09/2024 | 481 |
| Ornitela | 231635 | M | Hampshire | 27/03/2023 | 12/09/2024 | 536 |
| Ornitela | 231637 | F | Lancashire | 16/05/2023 | 11/09/2024 | 485 |
| Ornitela | 231639 | M | Lancashire | 16/05/2023 | 13/09/2024 | 487 |
| Ornitela | 231640 | F | Northumberland | 21/05/2023 | 20/06/2024 | 381 |
| Ornitela | 234264 | F | Hampshire | 10/05/2024 | 11/09/2024 | 125 |
| Ornitela | 234267 | F | Aberdeenshire | 06/06/2024 | 12/09/2024 | 99 |
| Ornitela | 234270 | F | County Durham | 29/05/2024 | 10/09/2024 | 105 |
| Pathtrack | 15569 | M | Aberdeenshire | 23/05/2019 | 05/07/2020 | 396 |
| Pathtrack | 15573 | M | Hampshire | 11/01/2019 | 21/07/2020 | 556 |
| Pathtrack | 15615 | M | Devon | 07/11/2021 | 08/08/2023 | 639 |
| Pathtrack | 15686 | F | Devon | 30/01/2020 | 13/04/2020 | 72 |
| Pathtrack | 15687 | M | Devon | 07/11/2021 | 22/05/2022 | 194 |
| Pathtrack | 15690 | M | Aberdeenshire | 01/06/2021 | 07/05/2023 | 605 |
| Pathtrack | 15696 | M | Devon | 07/11/2021 | 16/10/2022 | 344 |
| Pathtrack | 15702 | M | Devon | 07/11/2021 | 05/07/2022 | 241 |
| Pathtrack | 15725 | F | Devon | 07/11/2021 | 13/04/2022 | 158 |
| Pathtrack | 15745 | M | Aberdeenshire | 31/05/2021 | 01/08/2021 | 58 |
| Pathtrack | 15772 | M | Hampshire | 11/05/2021 | 17/08/2021 | 99 |
| Pathtrack | 15785 | F | Devon | 07/11/2021 | 15/08/2022 | 280 |
| Pathtrack | 15789 | F | Hampshire | 29/05/2020 | 24/07/2020 | 57 |
| Pathtrack | 42286 | M | Anglesey | 13/12/2020 | 29/07/2021 | 229 |
| Pathtrack | 42299 | M | Anglesey | 10/01/2022 | 09/02/2022 | 31 |

Table S2: Variables used to estimate fuel loads required for the first migratory flight and total migration duration in Eurasian Curlews using an adapted version of Pennycuick’s flight-range model [1]. To estimate migration costs, data on wing span, wing area, body mass, fat fraction, and muscle fraction were required (Table S2). Sex-specific morphological parameters were derived from a combination of empirical measurements and published sources. Total body mass was measured for each individual at capture. Wing span and wing area were obtained from species-level biometric data published by the British Trust for Ornithology. Muscle fraction was taken from data on closely related Icelandic Whimbrels (*Numenius phaeopus islandicus*) [2]. Fat fraction was estimated as the fraction of body mass lost between the heaviest individual prior to pre-breeding migration departure and the lightest individual upon arrival after pre-breeding migration. The heaviest individual at wintering sites was assumed to represent fully fuelled pre-migratory mass and the lightest individual at breeding grounds was assumed to represent lean mass post-migration. Lean body mass was calculated by subtracting fat mass from total body mass. The distance of the first migratory leg was derived from individual tracking data and converted to metres prior to analysis. Models were implemented in the *afpt* package [3] in R using default parameters described by Pennycuick and Battley [4], with an empirical scaling factor applied to represent typical migratory flight speeds of large waders. Air density was set to sea-level conditions at departure, and cruising altitude was set to 1500 m. First fuelling duration was estimated by dividing the required fuel load by a constant fuel deposition rate of 1.78% of lean body mass per day, representing an upper bound reported for migratory waders under favourable stopover conditions[2].

| Parameter |  | Females | N | Males | N |
| --- | --- | --- | --- | --- | --- |
| Wing span (m) |  | 0.923 | - | 0.877 | - |
| Wing area (m2) |  | 0.107 | - | 0.096 | - |
| Body mass (kg) | Mean | 0.842 | 55 | 0.700 | 54 |
|  | Maximum | 0.916 | 1 | 0.855 | 1 |
|  | Minimum | 0.722 | 1 | 0.582 | 1 |
| Fat fraction |  | 0.212 | - | 0.319 | - |
| Muscle fraction |  | 0.18 | 2 | 0.18 | 2 |
| Cruising altitude (m) |  | 1500 | - | 1500 | - |

Table S3: Results of LMMs examining variables influencing (a) departure dates, (b) arrival dates, (c) total distance travelled (km), (d) duration (d) and speed (km/d) during pre-breeding migration. Total distance and duration were log-transformed prior to modelling. Provided are sample sizes (N) and fixed effects with reference categories, estimates, standard errors (SE), t values, degrees of freedom (DF), chi-square (χ^2^) values, P values, proportion of variance explained by the fixed effects (R^2^_m)_ and by the random (individual identity) and fixed effects (R^2^_c_) of the final model. Statistics and P values are derived from Wald tests based on χ^2^ values. Significant P values are marked in bold (threshold ≤ 0.05). All continuous dependent variables are z-transformed to a mean of 0 and a standard deviation of 1.

| Parameter | N | Fixed effects | Reference | Estimate | ± SE | t value | DF | *χ2* value | *P* value | R2m | R2c |
| --- | --- | --- | --- | --- | --- | --- | --- | --- | --- | --- | --- |
| (a) Departure dates | 67 | Intercept |  | 60.844 | 6.454 | 9.427 |  |  |  | 0.321 | 0.906 |
|  |  | Sex | Female | -7.015 | 2.999 | -2.339 | 1 | 5.470 | **0.019** |  |  |
|  |  | Latitude |  | -2.131 | 1.518 | -1.404 | 1 | 1.972 | 0.160 |  |  |
|  |  | Temperature |  | -7.853 | 2.355 | -3.334 | 1 | 11.118 | **<0.001** |  |  |
|  |  | Precipitation |  | 0.529 | 0.908 | 0.583 | 1 | 0.340 | 0.560 |  |  |
|  |  | Wind speed |  | -0.878 | 1.189 | -0.738 | 1 | 0.545 | 0.460 |  |  |
|  |  | Wind direction |  | -0.403 | 0.931 | -0.433 | 1 | 0.187 | 0.665 |  |  |
|  |  | Year | 2019 |  |  |  | 5 | 37.469 | **<0.001** |  |  |
|  |  | 2020 |  | 3.393 | 4.834 | 0.702 |  |  |  |  |  |
|  |  | 2021 |  | -19.771 | 6.396 | -3.091 |  |  |  |  |  |
|  |  | 2022 |  | -4.944 | 5.909 | -0.837 |  |  |  |  |  |
|  |  | 2023 |  | -4.570 | 6.010 | -0.760 |  |  |  |  |  |
|  |  | 2024 |  | -9.633 | 5.921 | -1.627 |  |  |  |  |  |
|  |  | Latitude*Sex | Female | -2.905 | 3.966 | -0.732 | 1 | 0.536 | 0.464 |  |  |
|  |  | Temperature*Sex | Female | 7.571 | 5.407 | 1.400 | 1 | 1.960 | 0.161 |  |  |
|  |  | Precipitation*Sex | Female | -0.108 | 2.070 | -0.052 | 1 | 0.003 | 0.959 |  |  |
|  |  | Wind speed*Sex | Female | -2.016 | 2.213 | -0.911 | 1 | 0.829 | 0.362 |  |  |
|  |  | Wind direction*Sex | Female | -2.079 | 2.430 | -0.855 | 1 | 0.732 | 0.392 |  |  |
| (b) Arrival dates | 67 | Intercept |  | 50.568 | 10.530 | 4.802 |  |  |  | 0.220 | 0.734 |
|  |  | Sex | Female | -5.758 | 3.766 | -1.529 | 1 | 2.338 | 0.126 |  |  |
|  |  | Latitude |  | -1.299 | 1.950 | -0.666 | 1 | 0.444 | 0.505 |  |  |
|  |  | Temperature |  | -12.161 | 3.740 | -3.252 | 1 | 10.573 | **0.001** |  |  |
|  |  | Precipitation |  | 2.421 | 1.364 | 1.775 | 1 | 3.150 | 0.076 |  |  |
|  |  | Wind speed |  | 0.578 | 1.944 | 0.297 | 1 | 0.088 | 0.766 |  |  |
|  |  | Wind direction |  | -0.599 | 1.467 | -0.408 | 1 | 0.167 | 0.683 |  |  |
|  |  | Year | 2019 |  |  |  | 5 | 12.836 | **0.025** |  |  |
|  |  | 2020 |  | 4.202 | 9.393 | 0.447 |  |  |  |  |  |
|  |  | 2021 |  | 6.908 | 11.730 | 0.589 |  |  |  |  |  |
|  |  | 2022 |  | -5.768 | 10.434 | -0.553 |  |  |  |  |  |
|  |  | 2023 |  | 3.737 | 10.271 | 0.364 |  |  |  |  |  |
|  |  | 2024 |  | -3.901 | 10.099 | -0.386 |  |  |  |  |  |
|  |  | Latitude*Sex | Female | -2.283 | 4.866 | -0.469 | 1 | 0.220 | 0.639 |  |  |
|  |  | Temperature*Sex | Female | 1.547 | 9.245 | 0.167 | 1 | 0.028 | 0.867 |  |  |
|  |  | Precipitation*Sex | Female | 0.797 | 3.965 | 0.201 | 1 | 0.040 | 0.841 |  |  |
|  |  | Wind speed*Sex | Female | -1.507 | 3.225 | -0.467 | 1 | 0.219 | 0.640 |  |  |
|  |  | Wind direction*Sex | Female | -0.839 | 3.778 | -0.222 | 1 | 0.049 | 0.824 |  |  |
| (c) Distance | 67 | Intercept |  | 4.195 | 0.609 | 6.884 |  |  |  | 0.309 | 0.996 |
|  |  | Sex | Female | -0.378 | 0.422 | -0.896 | 1 | 0.702 | 0.402 |  |  |
|  |  | Latitude |  | 0.699 | 0.212 | 3.300 | 1 | 10.892 | **<0.001** |  |  |
|  |  | Temperature |  | -0.040 | 0.090 | -0.443 | 1 | 0.196 | 0.658 |  |  |
|  |  | Precipitation |  | -0.013 | 0.030 | -0.425 | 1 | 0.181 | 0.671 |  |  |
|  |  | Wind speed |  | 0.031 | 0.048 | 0.637 | 1 | 0.406 | 0.524 |  |  |
|  |  | Wind direction |  | -0.136 | 0.047 | -2.928 | 1 | 1.007 | 0.316 |  |  |
|  |  | Year | 2019 |  |  |  | 5 | 529.414 | **<0.001** |  |  |
|  |  | 2020 |  | 0.050 | 0.137 | 0.368 |  |  |  |  |  |
|  |  | 2021 |  | 3.002 | 0.187 | 16.022 |  |  |  |  |  |
|  |  | 2022 |  | 1.114 | 0.587 | 1.899 |  |  |  |  |  |
|  |  | 2023 |  | 1.211 | 0.580 | 2.087 |  |  |  |  |  |
|  |  | 2024 |  | 1.196 | 0.580 | 2.061 |  |  |  |  |  |
|  |  | Latitude*Sex | Female | 0.125 | 0.478 | 0.263 | 1 | 0.069 | 0.793 |  |  |
|  |  | Temperature*Sex | Female | -0.145 | 0.759 | -0.191 | 1 | 0.037 | 0.848 |  |  |
|  |  | Precipitation*Sex | Female | -0.093 | 0.076 | -1.230 | 1 | 1.512 | 0.219 |  |  |
|  |  | Wind speed*Sex | Female | -0.058 | 0.121 | -0.478 | 1 | 0.229 | 0.633 |  |  |
|  |  | Wind direction*Sex | Female | 0.151 | 0.053 | 2.825 | 1 | 7.979 | **0.005** |  |  |
| (d) Duration | 67 | Intercept |  | 1.697 | 0.431 | 3.940 |  |  |  | 0.337 | 0.939 |
|  |  | Sex | Female | -0.068 | 0.272 | -0.250 | 1 | 0.062 | 0.803 |  |  |
|  |  | Latitude |  | 0.438 | 0.129 | 3.389 | 1 | 11.487 | **<0.001** |  |  |
|  |  | Temperature |  | -0.035 | 0.226 | -0.157 | 1 | 0.025 | 0.876 |  |  |
|  |  | Precipitation |  | 0.114 | 0.062 | 1.828 | 1 | 3.342 | 0.068 |  |  |
|  |  | Wind speed |  | 0.080 | 0.093 | 0.862 | 1 | 0.742 | 0.389 |  |  |
|  |  | Wind direction |  | -0.018 | 0.071 | -0.261 | 1 | 0.068 | 0.794 |  |  |
|  |  | Year | 2019 |  |  |  | 5 | 96.392 | **<0.001** |  |  |
|  |  | 2020 |  | 0.026 | 0.341 | 0.076 |  |  |  |  |  |
|  |  | 2021 |  | 2.595 | 0.460 | 5.640 |  |  |  |  |  |
|  |  | 2022 |  | 0.006 | 0.431 | 0.015 |  |  |  |  |  |
|  |  | 2023 |  | 0.745 | 0.440 | 1.692 |  |  |  |  |  |
|  |  | 2024 |  | 0.561 | 0.442 | 1.269 |  |  |  |  |  |
|  |  | Latitude*Sex | Female | 0.031 | 0.334 | 0.092 | 1 | 0.009 | 0.926 |  |  |
|  |  | Temperature*Sex | Female | -0.143 | 0.607 | -0.236 | 1 | 0.056 | 0.813 |  |  |
|  |  | Precipitation*Sex | Female | -0.051 | 0.177 | -0.290 | 1 | 0.084 | 0.772 |  |  |
|  |  | Wind speed*Sex | Female | 0.037 | 0.197 | 0.188 | 1 | 0.035 | 0.851 |  |  |
|  |  | Wind direction*Sex | Female | 0.162 | 0.140 | 1.158 | 1 | 1.341 | 0.247 |  |  |
| (e) Speed | 67 | Intercept |  | 25.926 | 4.108 | 6.311 |  |  |  | 0.325 | 0.944 |
|  |  | Sex | Female | -3.170 | 2.499 | -1.269 | 1 | 1.610 | 0.205 |  |  |
|  |  | Latitude |  | 3.770 | 1.227 | 3.072 | 1 | 9.440 | **0.002** |  |  |
|  |  | Temperature |  | 4.410 | 1.615 | 2.730 | 1 | 7.453 | **0.006** |  |  |
|  |  | Precipitation |  | -0.770 | 0.529 | -1.455 | 1 | 2.118 | 0.146 |  |  |
|  |  | Wind speed |  | 1.049 | 0.720 | 1.457 | 1 | 2.123 | 0.145 |  |  |
|  |  | Wind direction |  | 0.147 | 0.489 | 0.300 | 1 | 0.090 | 0.764 |  |  |
|  |  | Year | 2019 |  |  |  | 5 | 27.983 | **<0.001** |  |  |
|  |  | 2020 |  | 2.111 | 3.025 | 0.698 |  |  |  |  |  |
|  |  | 2021 |  | 2.800 | 4.086 | 0.685 |  |  |  |  |  |
|  |  | 2022 |  | 5.807 | 3.884 | 1.495 |  |  |  |  |  |
|  |  | 2023 |  | -3.045 | 4.029 | -0.756 |  |  |  |  |  |
|  |  | 2024 |  | -3.587 | 3.977 | -0.902 |  |  |  |  |  |
|  |  | Latitude*Sex | Female | 1.517 | 2.919 | 0.520 | 1 | 0.270 | 0.603 |  |  |
|  |  | Temperature*Sex | Female | -0.447 | 5.630 | -0.079 | 1 | 0.006 | 0.937 |  |  |
|  |  | Precipitation*Sex | Female | -0.955 | 0.931 | -1.026 | 1 | 1.052 | 0.305 |  |  |
|  |  | Wind speed*Sex | Female | 1.763 | 1.282 | 1.375 | 1 | 1.892 | 0.169 |  |  |
|  |  | Wind direction*Sex | Female | -0.051 | 1.117 | -0.046 | 1 | 0.002 | 0.964 |  |  |

Table S4: Results of LMMs examining variables influencing (a) departure dates, (b) arrival dates, (c) total distance travelled (km), (d) duration (d) and speed (km/d) during post-breeding migration. Total distance and duration were log-transformed prior to modelling. Provided are sample sizes (N) and fixed effects with reference categories, estimates, standard errors (SE), t values, degrees of freedom (DF), chi-square (χ^2^) values, P values, proportion of variance explained by the fixed effects (R^2^_m)_ and by the random (individual identity) and fixed effects (R^2^_c_) of the final model. Statistics and P values are derived from Wald tests based on χ^2^ values. Significant P values are marked in bold (threshold ≤ 0.05). All continuous dependent variables are z-transformed to a mean of 0 and a standard deviation of 1.

| Parameter | N | Fixed effects | Reference | Estimate | ± SE | t value | DF | *χ2* value | *P* value | R2m | R2c |
| --- | --- | --- | --- | --- | --- | --- | --- | --- | --- | --- | --- |
| (a) Departure dates | 120 | Intercept |  | 161.193 | 2.415 | 66.737 |  |  |  | 0.579 | 0.706 |
|  |  | Sex | Female | 12.402 | 3.254 | 3.812 | 1 | 7.526 | **0.006** |  |  |
|  |  | Latitude |  | 7.719 | 1.239 | 6.232 | 1 | 38.835 | **<0.001** |  |  |
|  |  | Temperature |  | 17.392 | 2.795 | 6.223 | 1 | 42.305 | **<0.001** |  |  |
|  |  | Precipitation |  | 4.414 | 1.243 | 3.551 | 1 | 12.606 | **<0.001** |  |  |
|  |  | Wind speed |  | -1.779 | 1.232 | -1.445 | 1 | 2.087 | 0.149 |  |  |
|  |  | Wind direction |  | 2.307 | 1.010 | 2.285 | 1 | 5.221 | **0.022** |  |  |
|  |  | Year | 2018 |  |  |  | 6 | 5.876 | 0.437 |  |  |
|  |  | 2019 |  | 1.653 | 8.293 | 0.199 |  |  |  |  |  |
|  |  | 2020 |  | -3.015 | 7.883 | -0.382 |  |  |  |  |  |
|  |  | 2021 |  | 3.622 | 7.203 | 0.503 |  |  |  |  |  |
|  |  | 2022 |  | -2.666 | 5.875 | -0.454 |  |  |  |  |  |
|  |  | 2023 |  | -2.882 | 5.708 | -0.505 |  |  |  |  |  |
|  |  | 2024 |  | 1.944 | 6.007 | 0.324 |  |  |  |  |  |
|  |  | Latitude*Sex | Female | 1.788 | 2.669 | 0.670 | 1 | 0.449 | 0.503 |  |  |
|  |  | Temperature*Sex | Female | -9.354 | 3.498 | -2.674 | 1 | 7.150 | **0.007** |  |  |
|  |  | Precipitation*Sex | Female | 2.962 | 2.530 | 1.171 | 1 | 1.371 | 0.242 |  |  |
|  |  | Wind speed*Sex | Female | -3.989 | 2.792 | -1.429 | 1 | 2.041 | 0.153 |  |  |
|  |  | Wind direction*Sex | Female | -1.897 | 2.022 | -0.938 | 1 | 0.880 | 0.348 |  |  |
| (b) Arrival dates | 120 | Intercept |  | 176.563 | 3.162 | 55.830 |  |  |  | 0.280 | 0.570 |
|  |  | Sex | Female | 9.917 | 5.153 | 1.925 | 1 | 3.704 | 0.054 |  |  |
|  |  | Latitude |  | 6.760 | 2.491 | 2.714 | 1 | 7.364 | **0.007** |  |  |
|  |  | Temperature |  | 10.326 | 3.567 | 2.894 | 1 | 8.378 | **0.004** |  |  |
|  |  | Precipitation |  | 10.751 | 2.350 | 4.575 | 1 | 20.926 | **<0.001** |  |  |
|  |  | Wind speed |  | -5.845 | 2.460 | -2.376 | 1 | 5.648 | **0.017** |  |  |
|  |  | Wind direction |  | 2.173 | 1.957 | 1.111 | 1 | 1.233 | 0.267 |  |  |
|  |  | Year | 2018 |  |  |  | 6 | 1.440 | 0.963 |  |  |
|  |  | 2019 |  | -0.368 | 16.641 | -0.022 |  |  |  |  |  |
|  |  | 2020 |  | -5.613 | 15.958 | -0.352 |  |  |  |  |  |
|  |  | 2021 |  | -1.200 | 14.412 | -0.083 |  |  |  |  |  |
|  |  | 2022 |  | 5.093 | 11.789 | 0.432 |  |  |  |  |  |
|  |  | 2023 |  | 1.242 | 11.454 | 0.108 |  |  |  |  |  |
|  |  | 2024 |  | 0.454 | 12.040 | 0.038 |  |  |  |  |  |
|  |  | Latitude*Sex | Female | 2.572 | 5.124 | 0.502 | 1 | 0.252 | 0.616 |  |  |
|  |  | Temperature*Sex | Female | -1.102 | 7.436 | -0.148 | 1 | 0.022 | 0.882 |  |  |
|  |  | Precipitation*Sex | Female | 8.444 | 5.099 | 1.656 | 1 | 2.743 | 0.098 |  |  |
|  |  | Wind speed*Sex | Female | -0.861 | 5.622 | -0.153 | 1 | 0.024 | 0.878 |  |  |
|  |  | Wind direction*Sex | Female | 0.367 | 4.219 | 0.087 | 1 | 0.008 | 0.931 |  |  |
| (c) Distance | 120 | Intercept |  | 5.004 | 0.119 | 42.056 |  |  |  | 0.249 | 0.914 |
|  |  | Sex | Female | -0.215 | 0.260 | -0.826 | 1 | 0.683 | 0.409 |  |  |
|  |  | Latitude |  | 0.594 | 0.118 | 5.028 | 1 | 25.283 | **<0.001** |  |  |
|  |  | Temperature |  | -0.032 | 0.116 | -0.277 | 1 | 0.077 | 0.782 |  |  |
|  |  | Precipitation |  | 0.007 | 0.058 | 0.123 | 1 | 0.015 | 0.902 |  |  |
|  |  | Wind speed |  | -0.055 | 0.068 | -0.807 | 1 | 0.652 | 0.420 |  |  |
|  |  | Wind direction |  | 0.052 | 0.056 | 0.922 | 1 | 0.850 | 0.357 |  |  |
|  |  | Year | 2018 |  |  |  | 6 | 6.482 | 0.371 |  |  |
|  |  | 2019 |  | -0.052 | 0.613 | -0.085 |  |  |  |  |  |
|  |  | 2020 |  | 0.585 | 0.618 | 0.947 |  |  |  |  |  |
|  |  | 2021 |  | 0.295 | 0.596 | 0.495 |  |  |  |  |  |
|  |  | 2022 |  | 0.465 | 0.542 | 0.858 |  |  |  |  |  |
|  |  | 2023 |  | 0.371 | 0.540 | 0.687 |  |  |  |  |  |
|  |  | 2024 |  | 0.326 | 0.542 | 0.602 |  |  |  |  |  |
|  |  | Latitude*Sex | Female | -0.069 | 0.253 | -0.274 | 1 | 0.075 | 0.784 |  |  |
|  |  | Temperature*Sex | Female | -0.259 | 0.180 | -1.440 | 1 | 2.075 | 0.150 |  |  |
|  |  | Precipitation*Sex | Female | 0.234 | 0.129 | 1.819 | 1 | 3.308 | 0.069 |  |  |
|  |  | Wind speed*Sex | Female | -0.043 | 0.157 | -0.276 | 1 | 0.076 | 0.782 |  |  |
|  |  | Wind direction*Sex | Female | 0.045 | 0.107 | 0.420 | 1 | 0.177 | 0.674 |  |  |
| (d) Duration | 120 | Intercept |  | 2.163 | 0.095 | 22.670 |  |  |  | 0.172 | 0.725 |
|  |  | Sex | Female | 0.060 | 0.215 | 0.280 | 1 | 0.079 | 0.779 |  |  |
|  |  | Latitude |  | 0.391 | 0.095 | 4.110 | 1 | 16.890 | **<0.001** |  |  |
|  |  | Temperature |  | -0.059 | 0.138 | -0.424 | 1 | 0.180 | 0.672 |  |  |
|  |  | Precipitation |  | 0.098 | 0.074 | 1.322 | 1 | 1.747 | 0.186 |  |  |
|  |  | Wind speed |  | -0.109 | 0.081 | -1.340 | 1 | 1.794 | 0.180 |  |  |
|  |  | Wind direction |  | -0.012 | 0.071 | -0.176 | 1 | 0.031 | 0.860 |  |  |
|  |  | Year | 2018 |  |  |  | 6 | 8.739 | 0.189 |  |  |
|  |  | 2019 |  | -0.319 | 0.616 | -0.518 |  |  |  |  |  |
|  |  | 2020 |  | 0.158 | 0.573 | 0.276 |  |  |  |  |  |
|  |  | 2021 |  | -0.291 | 0.538 | -0.542 |  |  |  |  |  |
|  |  | 2022 |  | 0.362 | 0.454 | 0.796 |  |  |  |  |  |
|  |  | 2023 |  | 0.222 | 0.450 | 0.494 |  |  |  |  |  |
|  |  | 2024 |  | 0.051 | 0.452 | 0.114 |  |  |  |  |  |
|  |  | Latitude*Sex | Female | -0.069 | 0.209 | -0.329 | 1 | 0.108 | 0.742 |  |  |
|  |  | Temperature*Sex | Female | 0.007 | 0.233 | 0.028 | 1 | 0.001 | 0.978 |  |  |
|  |  | Precipitation*Sex | Female | 0.311 | 0.168 | 1.851 | 1 | 3.427 | 0.064 |  |  |
|  |  | Wind speed*Sex | Female | -0.090 | 0.185 | -0.485 | 1 | 0.235 | 0.628 |  |  |
|  |  | Wind direction*Sex | Female | 0.081 | 0.130 | 0.623 | 1 | 0.388 | 0.534 |  |  |
| (e) Speed | 120 | Intercept |  | 18.615 | 3.487 | 5.339 |  |  |  | 0.213 | 0.637 |
|  |  | Sex | Female | -4.915 | 1.585 | -3.100 | 1 | 5.450 | **0.020** |  |  |
|  |  | Latitude |  | 4.213 | 0.774 | 5.441 | 1 | 24.957 | **<0.001** |  |  |
|  |  | Temperature |  | 1.385 | 1.083 | 1.279 | 1 | 1.636 | 0.201 |  |  |
|  |  | Precipitation |  | -1.870 | 0.645 | -2.900 | 1 | 4.458 | **0.035** |  |  |
|  |  | Wind speed |  | 1.089 | 0.641 | 1.699 | 1 | 2.885 | 0.089 |  |  |
|  |  | Wind direction |  | 0.427 | 0.512 | 0.835 | 1 | 0.697 | 0.404 |  |  |
|  |  | Year | 2018 |  |  |  | 6 | 10.681 | 0.099 |  |  |
|  |  | 2019 |  | -0.979 | 4.703 | -0.208 |  |  |  |  |  |
|  |  | 2020 |  | 2.145 | 4.337 | 0.495 |  |  |  |  |  |
|  |  | 2021 |  | 8.108 | 4.027 | 2.013 |  |  |  |  |  |
|  |  | 2022 |  | 1.352 | 3.398 | 0.398 |  |  |  |  |  |
|  |  | 2023 |  | 3.501 | 3.333 | 1.051 |  |  |  |  |  |
|  |  | 2024 |  | 2.321 | 3.405 | 0.682 |  |  |  |  |  |
|  |  | Latitude*Sex | Female | 0.123 | 1.533 | 0.080 | 1 | 0.006 | 0.936 |  |  |
|  |  | Temperature*Sex | Female | -3.150 | 1.729 | -1.822 | 1 | 3.320 | 0.068 |  |  |
|  |  | Precipitation*Sex | Female | -0.584 | 1.331 | -0.439 | 1 | 0.193 | 0.661 |  |  |
|  |  | Wind speed*Sex | Female | -1.493 | 1.445 | -1.033 | 1 | 1.068 | 0.301 |  |  |
|  |  | Wind direction*Sex | Female | 0.547 | 1.043 | 0.524 | 1 | 0.275 | 0.600 |  |  |

Table S5: Results of LMMs examining breeding success (failure versus success) and previously identified significant effects on (a) departure dates, (b) arrival dates, (c) total distance travelled (km), (d) duration (d) and speed (km/d) during post-breeding migration. Total distance and duration were log-transformed prior to modelling. Provided are sample sizes (N) and fixed effects with reference categories, estimates, standard errors (SE), t values, degrees of freedom (DF), chi-square (χ^2^) values, P values, proportion of variance explained by the fixed effects (R^2^_m)_ and by the random (individual identity) and fixed effects (R^2^_c_). Statistics and P values are derived from Wald tests based on χ^2^ values. Significant P values are marked in bold (threshold ≤ 0.05). All continuous dependent variables are z-transformed to a mean of 0 and a standard deviation of 1.

| Parameter | N | Fixed effects | Reference | Estimate | ± SE | t value | DF | *χ2* value | *P* value | R2m | R2c |
| --- | --- | --- | --- | --- | --- | --- | --- | --- | --- | --- | --- |
| (a) Departure dates | 85 | Intercept |  | 166.046 | 3.125 | 53.138 |  |  |  | 0.613 | 0.711 |
|  |  | Breeding success | No | 8.004 | 2.916 | 2.745 | 1 | 6.976 | **0.008** |  |  |
|  |  | Sex | Female | 7.208 | 3.348 | 2.153 | 1 | 4.634 | **0.031** |  |  |
|  |  | Latitude |  | 8.619 | 1.929 | 4.467 | 1 | 12.000 | **0.001** |  |  |
|  |  | Temperature |  | 6.682 | 2.675 | 2.498 | 1 | 6.239 | **0.012** |  |  |
|  |  | Precipitation |  | 5.864 | 1.505 | 3.896 | 1 | 15.178 | **<0.001** |  |  |
|  |  | Wind direction |  | 2.385 | 1.208 | 1.975 | 1 | 3.899 | **0.048** |  |  |
|  |  | Temperature*Sex | Female | -6.569 | 5.078 | -1.294 | 1 | 1.673 | 0.196 |  |  |
|  |  | Breeding success*Sex | No/Female | 8.202 | 6.654 | 1.233 | 1 | 1.519 | 0.218 |  |  |
|  |  | Breeding success*Latitude | No | -6.975 | 2.442 | -2.856 | 1 | 8.159 | **0.004** |  |  |
| (b) Arrival dates | 85 | Intercept |  | 179.395 | 6.666 | 26.913 |  |  |  | 0.324 | 0.723 |
|  |  | Breeding success | No | 2.444 | 5.976 | 0.409 | 1 | 0.167 | 0.683 |  |  |
|  |  | Sex | Female | 4.941 | 7.316 | 0.675 | 1 | 0.456 | 0.499 |  |  |
|  |  | Latitude |  | 3.258 | 3.525 | 0.924 | 1 | 0.854 | 0.355 |  |  |
|  |  | Temperature |  | 2.515 | 5.308 | 0.474 | 1 | 0.225 | 0.636 |  |  |
|  |  | Precipitation |  | 14.264 | 2.767 | 5.155 | 1 | 26.579 | **<0.001** |  |  |
|  |  | Wind speed |  | -10.480 | 3.113 | -3.367 | 1 | 11.334 | **<0.001** |  |  |
|  |  | Breeding success*Sex | No/Female | 17.562 | 13.601 | 1.291 | 1 | 1.667 | 0.197 |  |  |
|  |  | Breeding success*Latitude | No | -3.824 | 4.867 | -0.786 | 1 | 0.617 | 0.432 |  |  |
| (c) Distance | 85 | Intercept |  | 5.306 | 0.286 | 18.578 |  |  |  | 0.281 | 0.950 |
|  |  | Breeding success | No | -0.098 | 0.120 | -0.819 | 1 | 0.671 | 0.413 |  |  |
|  |  | Sex | Female | -0.514 | 0.335 | -1.534 | 1 | 2.354 | 0.125 |  |  |
|  |  | Latitude |  | 0.696 | 0.142 | 4.902 | 1 | 24.032 | **<0.001** |  |  |
|  |  | Breeding success*Sex | No/Female | -0.234 | 0.260 | -0.900 | 1 | 0.811 | 0.368 |  |  |
|  |  | Breeding success*Latitude | No | 0.156 | 0.258 | 0.605 | 1 | 0.366 | 0.545 |  |  |
| (d) Duration | 85 | Intercept |  | 2.319 | 0.255 | 9.085 |  |  |  | 0.179 | 0.761 |
|  |  | Breeding success | No | 0.003 | 0.179 | 0.017 | 1 | 0.000 | 0.987 |  |  |
|  |  | Sex | Female | -0.260 | 0.287 | -0.903 | 1 | 0.816 | 0.366 |  |  |
|  |  | Latitude |  | 0.435 | 0.124 | 3.498 | 1 | 12.234 | **<0.001** |  |  |
|  |  | Breeding success*Sex | No/Female | -0.110 | 0.532 | -0.206 | 1 | 0.043 | 0.836 |  |  |
|  |  | Breeding success*Latitude | No | 0.121 | 0.175 | 0.690 | 1 | 0.476 | 0.490 |  |  |
| (e) Speed | 85 | Intercept |  | 20.163 | 1.783 | 11.312 |  |  |  | 0.246 | 0.669 |
|  |  | Breeding success | No | 1.071 | 1.387 | 0.772 | 1 | 0.596 | 0.440 |  |  |
|  |  | Sex | Female | -4.052 | 1.971 | -2.056 | 1 | 4.226 | **0.040** |  |  |
|  |  | Latitude |  | 3.555 | 0.858 | 4.141 | 1 | 17.150 | **<0.001** |  |  |
|  |  | Precipitation |  | -1.408 | 0.753 | -1.869 | 1 | 3.492 | 0.062 |  |  |
|  |  | Breeding success*Sex | No/Female | -1.982 | 2.942 | -0.674 | 1 | 0.454 | 0.501 |  |  |
|  |  | Breeding success*Latitude | No | 0.499 | 1.656 | 0.302 | 1 | 0.091 | 0.763 |  |  |

**References**

1.	Pennycuick CJ. 2008 *Modelling the Flying Bird*. Amsterdam Boston Paris: Academic Press Inc.

2.	Carneiro C, Gunnarsson TG, Alves JA. 2019 Faster migration in autumn than in spring: seasonal migration patterns and non-breeding distribution of Icelandic whimbrels *Numenius phaeopus islandicus*. *J. Avian Biol.* **50**. (doi:10.1111/jav.01938)

3.	KleinHeerenbrink M, Hedenström A. 2023 afpt: Tools for Modelling of Animal Flight Performance.

4.	Pennycuick CJ, Battley PF. 2003 Burning the engine: a time-marching computation of fat and protein consumption in a 5420-km non-stop flight by great knots, *Calidris tenuirostris*. *Oikos* **103**, 323–332. (doi:10.1034/j.1600-0706.2003.12124.x)
